# Adaptation of the Ambulatory and Home Care Record for collecting palliative care service utilisation data from family carers in the UK: a pilot study

**DOI:** 10.1186/s40814-018-0332-2

**Published:** 2018-08-18

**Authors:** Laura M. Holdsworth, Heather Gage, Peter Williams, Claire Butler

**Affiliations:** 1 Primary Care and Population Health, Stanford University School of Medicine, 1265 Welch Road, MSOB, Stanford, CA 94305 USA; 2 Surrey Health Economics Centre, Department of Clinical and Experimental Medicine, University of Surrey, Guildford, GU2 7XH UK; 3 Department of Mathematics, Faculty of Engineering and Physical Sciences, University of Surrey, Guildford, GU2 7XH UK; 4 Centre for Health Services Studies, University of Kent, Canterbury, CT2 2NF UK

**Keywords:** Service utilisation, Palliative care, Questionnaire, Telephone interview, Pilot study, Informal care

## Abstract

**Background:**

Measuring service use and costs is an important aspect of service delivery evaluation. In end-of-life care, there is heavy reliance on care by family/friends (informal carers) and this should be reflected in the total cost of care alongside formal services. The Ambulatory and Home Care Record, developed in Canada, is both comprehensive in coverage and validated for collecting data on formal and informal caring. This study aimed to adapt and pilot the Ambulatory and Home Care Record questionnaire for use in the UK within a study evaluating a new palliative care service. The objectives were to test if family carers could be recruited and assess acceptability and usability of data gathered.

**Methods:**

Single cohort pilot study using a structured telephone questionnaire carried out every other week. Family carers of patients newly added to the palliative care register or referred to hospice services in the South East of England were invited to participate by mail. Volunteers remained in the study for a maximum of six interviews or until the patient died.

**Results:**

In total, 194 carers were invited by mail to participate in the study, of which 23 (11.8%) completed at least one interview and 16 (8.2%) completed all possible interviews. Recruitment to the study was lower than anticipated, but most participants seemed to find the interviews acceptable. The modified questionnaire produced usable and relevant data for an economic evaluation of formal and informal caring costs.

**Conclusions:**

Modifications are needed to the process of recruitment as a postal recruitment strategy did not have a high response rate. The Ambulatory and Home Care Record has proved a viable tool for use in the UK setting, with a few minor modifications, and will be used in a larger study comparing hospice models.

**Electronic supplementary material:**

The online version of this article (10.1186/s40814-018-0332-2) contains supplementary material, which is available to authorized users.

## Background

Measuring the costs of palliative and end-of-life care is challenging but is important for the evaluation of different service delivery models [1, 2]. Studies typically report that hospital stays and residential care represent the major cost of care, depending on the cause of death [3, 4]. However, when care provided by family carers (informal care) is included in cost analyses, hospital costs are replaced by more unpaid carer time and outpatient services for home death patients, and the resource implications of hospice and home become largely equivalent as places of death [5]. Early studies of palliative care have often not included all components of costs, particularly time and money provided by family carers [6, 7]. Capture of these items is challenging, and issues arise around valuing caregiver time [8, 9]. Various approaches have been used to try and assemble details of the formal and informal inputs in palliative care settings, including record reviews, modelling, structured questionnaires and interviews with family carers before and after the death of the patient. Amongst the primary data collection methods, only one instrument, the Ambulatory and Home Care Record (AHCR), has been found comprehensive in coverage and validated [10, 11].

The AHCR was first developed in Canada in 1997 to prospectively capture ambulatory and home services for economic evaluations and has been validated with cystic fibrosis patients [12]. It has subsequently been used in Canada for assessing the private costs of home care [13] and in studies of palliative care that include unpaid (informal) caring costs [14–16]. The AHCR involves interviews with family carers of patients at two weekly intervals, up to the point of death, to collect information on the services that have been received. This paper reports the findings from a pilot study that sought to adapt and test the AHCR for gathering information on palliative care costs in the United Kingdom (UK) as currently no suitable tool exists in the UK setting. Improvements to end-of-life care is a health policy priority in the UK which emphasises better coordination of services and enabling greater choice and control over the place of death for patients and carers [17]. A lack of information on end-of-life care costs and a need for more research on the resource implications of alternative models of care has been identified to help establish cost-effective service configurations and to inform service commissioning [17–23].

The AHCR was adapted and trialled as a pilot embedded in a study evaluating a new palliative care telephone navigation service offered by a major hospice provider in South East England. This was a pilot test of the AHCR’s acceptability to family carers and utility to researchers for gathering service-use data, in preparation for embarking on a large study comparing models of home palliative care [24]. The study aimed to explore whether a modified AHCR could be successfully used in the UK context for gathering information on formal and informal service use, from which costs of end-of-life care could be calculated. The specific objectives were to test:

1. Recruitment and retention of family carers to a study using the AHCR;
2. Acceptability of the adapted instrument and data collection process; and
3. Usability of the data for economic evaluation of palliative care models.

## Methods

### Design

The design of the pilot was a single cohort with no comparison group.

### Instrument adaptation

The AHCR was designed as a structured, paper-based instrument for self-administration or interview, on a two-weekly basis. It collects information from an informal carer about the care received by “the patient”. The questions are grouped around services received inside the home, outside the home (outpatient, emergency department), inpatient stays (hospital and hospice), telephone calls, medications, supplies and equipment and unpaid caregiving by family and friends (see Additional file 1). Background information about the patient and family carer is gathered in the first interview. Although the overall structure of the AHCR remained the same, small changes were made to suit the particular features of the UK health and social care system (Table 1).

**Table 1:** Ambulatory and Home Care Record (AHCR) adaptations made for a UK population

Original AHCR	UK AHCR (adapted)	Reason for change
Home visits, health care only	Home visits, health and social care	Most healthcare visits are paid for by public funds; social care is usually free at end-of-life if deemed medically necessary (otherwise means tested).
Length of home visits	Removed	Prior experience of research team indicated this would be poorly reported and because nationally validated data are available.^a^
Payment for home visits and reimbursement received	Removed	Prior experience of research team indicated this would be poorly reported and unlikely to be relevant since palliative care costs are covered free of charge by the National Health Service and voluntary sector.
Phone calls to palliative team only	Phone calls to any health and social service, not just palliative care team	Greater variability in local services in the UK; palliative care patients use the whole system of health and social care.
Questions about medications and costs	One generic question about whether exempt from prescription costs	People over 60 years and/or at a palliative stage (medical exemption) do not pay for items obtained on prescription in UK.
New question	Whether carer received Carer’s Allowance	Carer’s Allowance is a benefit available in UK.
New question	Whether services met (or fell short or exceeded expectations) was added	The pilot was being conducted in the context of the roll out of a new telephone navigation service and satisfaction with services was of interest to the provider.
Language and terminology	Minor changes, e.g. use of A&E instead of ER	To customise language and terminology to UK.

^a^Unit Costs of Health and Social Care, Personal and Social Sciences Research Unit. http://www.pssru.ac.uk/project-pages/unit-costs/

The service-use data that are captured provide a societal perspective on costs covering three categories: public, private and unpaid (informal) care costs [16]. In the UK, public costs relate to services financed by government from tax revenue (and some palliative care services provided by the voluntary sector): healthcare appointments; emergency department visits; laboratory and diagnostic tests; treatments; prescription medicines, supplies, equipment; inpatient care (hospital, hospice, nursing home); and home care services. Prescription medicines and some supplies are exempt from charges for palliative care patients. Private costs are all costs not publicly financed: out-of-pocket expenses on healthcare appointments, paid home carers, travel expenses, private insurance payments (if applicable), treatments bought privately, over-the-counter medications and co-payments for means tested items. Unpaid (or informal) care costs include the time devoted by family and friends to caring (both time lost from paid employment and from leisure or household tasks).

We also captured patient functional status using the Eastern Cooperative Oncology Group (ECOG) scale [25], carer burden [26] and views on service satisfaction as part of the telephone survey. These were not part of the AHCR but were warranted for other reasons; ECOG and carer burden because the scores may correlate with service use and informal caring; and service satisfaction because the provider of the new navigation service was interested in feedback.

### Sample and access

Potential participants were identified by primary care practice managers in South East England when patients were added to their palliative care registers and by a hospice data manager when patients were referred to the hospice. Letters of invitation to participate in the research, an information sheet, consent form and pre-paid reply envelope were sent to carers by post by the primary care practice and hospice. Where no carer was listed in the patient’s medical records, a letter was sent to the patient asking them to identify a carer who would then be sent an invitation by the researcher. Volunteers replied direct to an independent university researcher (LMH) who carried out the telephone survey. We aimed to recruit 30 carers which is considered a suitable sample size for a pilot study [27].

### Procedure

Telephone surveys were carried out until: the patient died, the end of the study period, or when six surveys (approximately 10 weeks) had been completed with the carer. If the patient had survived, the maximum number of surveys was set at six to avoid overburdening carers.

Survey interviews were recorded with carer permission for reference and responses were noted and transcribed into an Excel spreadsheet immediately following the interview. A narrative summary of the survey interview was added to the spreadsheet to aid analysis.

### Analysis

The recruitment rate was assessed by monitoring the number of referrals from primary care or the hospice, invitations sent and consents received. The number of completed surveys, relative to expected number (i.e. every 2 weeks between recruitment and patient death) was used to indicate retention of carers in the study. The reasons for withdrawal were explored, where possible.

Acceptability of the instrument and the two-weekly data collection process was assessed subjectively through experiences noted by the interviewer and objectively by recruitment and retention rates.

The usability of the data collected for the economic evaluation was assessed through descriptive analysis and inspection of data. The characteristics of participants were analysed descriptively, including ECOG performance status [25], carer burden scales [26] and service satisfaction.

Since participants were in the study for different periods of time, service-use data were standardised by calculating the number of contacts, for each service, over a 28-day period. Unit costs obtained from validated national sources [28] were applied to service use to calculate patient costs per 28 days and the distribution of expenditure on different categories of care. The number of hours per day reported as spent by the primary and secondary carers were converted to hours per 28 days and a replacement cost attributed based on a clinical support worker [28] (see Additional file 2). Costs were reported in British pounds using rates from 2016.

## Results

### Recruitment, retention and reasons for withdrawal

**Fig. 1:**
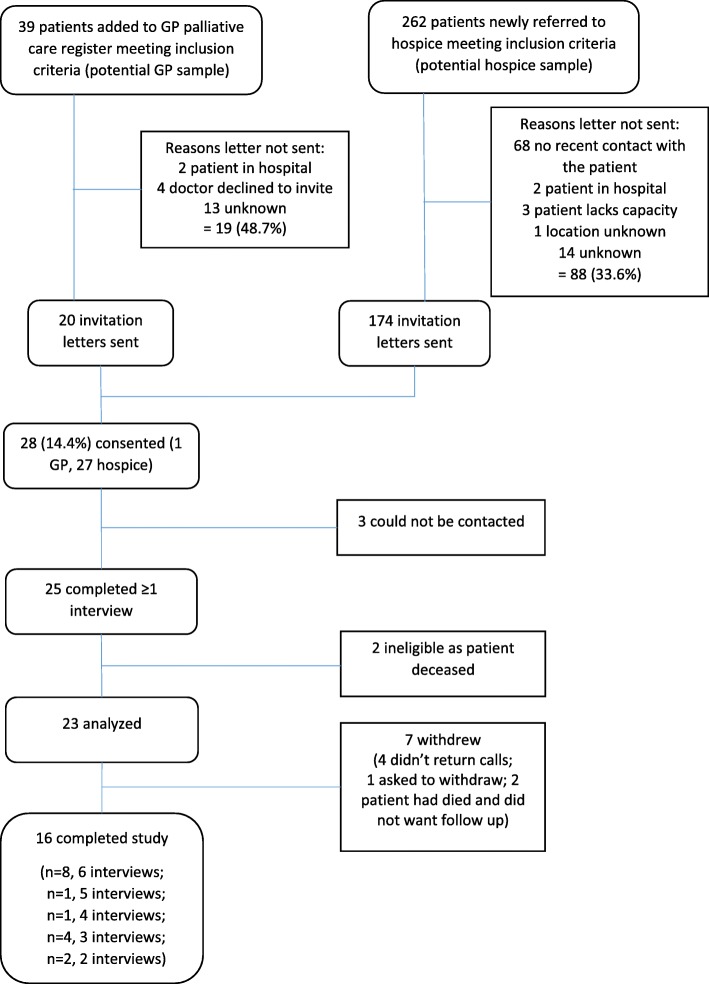
Recruitment and retention of carers

Recruitment and retention is shown in Fig. 1. Recruitment took place between August 2012 and February 2014 (with a break between September and December 2013 due to service changes), until sufficient data were collected to decide feasibility. Initially, six primary care practices had agreed to identify patients, but only one practice participated. Thirty-nine patients were added to the palliative care register from this practice, and 262 were newly referred to the hospice. More than one third (107, 35.5%) were not sent a letter of invitation to the study for multiple reasons, most commonly because the hospice had not had recent contact with the patient (68), or no reason was given (27). In total, 194 carers/patients were invited to participate over the 15-month recruitment period and 28 (14.4%) carers provided written consent. Three of these carers could not be contacted and never had a first interview. Two patients had died prior to the first interview with the carer and so were excluded. Twenty-three (11.9%) carers were included in the final analysis for each objective.

Sixteen carers (69.6%) completed the study (i.e. completed six interviews, or the patient died and the carer was interviewed after death). The carers who withdrew from the study (Table 2) either did not return telephone messages after three attempts (4 carers), or notified the researcher that they wished to withdraw due to feeling overwhelmed with patient care (1 carer), or the patient had died and the carer did not wish to complete an after death interview (2 carers). Eleven (47.8%) patients died by the end of the study and seven (63.6%) of those carers completed an interview following the patient’s death.

**Table 2:** Number of telephone survey interviews completed by carers and whether they completed all study surveys or withdrew, *n* = 23

Number of completed survey interviews	Number of carers	Carer completed study	Patients alive at last survey	Carer withdrew	Reason withdrew
1	2	–	–	2	1—did not return messages 1—requested withdrawal (too much going on)
2	3	2	–	1	Patient died, carer did not want follow-up
3	4	4	–	–	–
4	5	1	–	4	3—did not return messages 1—patient died, carer did not want follow-up
5	1	1	1	–	–
6	8	8	8	–	–
Total	23	16	9	7	

### Acceptability of the instrument and data collection procedure

Survey interviews were carried out approximately every 2 weeks. Even though some carers requested to reschedule to suit their needs, the average time between surveys was 15 days. Though the AHCR questions are structured, responses frequently took on unstructured characteristics as carers volunteered unsolicited information about their experiences thus accounting for a wide variation in survey interview length, anywhere from 3 min to 40 min, with most taking between 5 and 10 min. Several carers kept track of appointments in their diaries and used these to aid with answering questions. Interviews had a conversational style to encourage participants to give details of service use as these stories often helped them to recall and clarify service utilisation. This was particularly useful as carers sometimes forgot what services the patient had received. For example, a carer would say that they had not made any phone calls, but then while talking about a hospital admission, it would emerge that the carer had called urgent care and then emergency services for an ambulance, which led to the hospital admission. During such lengthy discussions, the researcher would interpret and agree with the carer a structured response whilst noting their additional comments.

### Usability and relevance of data

Interviews yielded complete and usable data on the background characteristics of participants (Table 3) and service use (Table 4). Most carers lived with the patient and were spouses, a quarter were in paid employment and one half were university educated. Carer burden increased closer to the death of the patient. The average age of patients was 70 years, and most had a diagnosis of cancer. In most cases, carers reported that services met or exceeded expectations. The only services used by more than 50% of patients were primary care, district nursing and hospital outpatient consultations; a small number (30%) used home care intensely, and hospital or hospice stays were the costliest items. Reported out-of-pocket expenditure was relatively low (average of £6.40/month). Many respondents reported receiving supplies and equipment on loan from health, social and voluntary services (Table 4).

**Table 3:** Characteristics of the carers and patients included in the analysis, *n* = 23

		*n* (%)
Carer (participant)		
Gender	Female	16 (69.6)
Age	Median (range)	63 (44–81)
Relationship to patient	Spouse/partner	17 (73.9)
	Child	4 (17.4)
	Parent	1 (4.3)
	Other	1 (4.3)
Lives with/staying with patient		21 (91.3)
In full or part time work (vs retired)		6 (26.0)
College or university educated		11 (47.8)
		Baseline; last recorded
Carer burden during end-of-life care [26], range 0 (best)–100 (worst)	Mean	34.78; 40.83
	Standard deviation	25.16; 26.89
	Median	29.17; 35.00
	Range	0–90; 0–100
	Best quartile, *n* %	11(47.8); 8(34.8)
	2nd quartile, *n* %	7(30.5); 9(39.1)
	3rd quartile, *n* %	3(13.0); 3(13.0)
	Worst quartile, *n* %	2(8.6); 3(13.0)
Patient		
Gender	Female	10 (43.5)
Age	Median (range)	70 (43–93)
Lives alone (vs with spouse/other)		3 (13.0)
College or university educated		9 (45.0)
Receives attendance allowance^a^		11 (47.8)
Diagnosis	Cancer	20 (87.0)
	Non-cancer	3 (13.0)
Place of death	Hospice	4 (17.4)
	Hospital	4 (17.4)
	Home	2 (8.7)
	Unknown or alive at end of study	13 (56.5)
		Baseline; last recorded
Functional status—ECOG [25]	0. Fully active	0; 0
	1. Restricted in strenuous activity	1(4.3); 2(8.7)
	2. Ambulatory, capable of self-care but not work	11(47.8); 5(21.7)
	3. Limited self-care	7(30.4); 8(34.8)
	4. Completely disabled	4(17.4); 3(13.0)
	5. Dead	0; 5(21.7)
Service satisfaction		Baseline; last recorded
	Exceeds expectations	10(43**.**5); 10(43.5)
	Meets expectations	10(43.5); 12(52.2)
	Falls short of expectations	3(13.0); 1(4.3)

^a^Benefit available in the UK

**Table 4:** Service use and costs reported by 23 carers, standardised to number of contacts/costs per 28 days

Category	Service	Service use						Costs (£, 2016)			
		Number with zero	% with zero	Mean	SD	Median	Range	Mean cost (£)	SD cost (£)	Median cost (£)	Cost range (£)
In home	GP visit	11	47.8	.705	1.16	.346	0–4.31	49.89	82.33	24.46	0 to 304.77
	District nurse	10	43.5	3.61	6.44	.66	0–26.96	98.62	175.92	17.99	0 to 736.09
	Hospice nurse	15	65.2	.372	.650	0	0–2.05	10.16	17.71	0	0 to 55.93
	Hospice outreach	21	91.3	.110	.417	0	0–1.93	3.01	11.37	0	0 to 52.72
	Home care	16	69.6	20.48	37.4	0	0–105.5	245.81	448.82	0	0 to 1266.46
	Counsellor	22	95.7	.017	.080	0	0–.38	0.70	3.36	0	0 to 16.11
	Physiotherapist	19	82.6	.373	1.35	0	0–6.41	8.21	29.70	0	0 to 141.01
	Occupational therapist	20	87.0	.095	.302	0	0–1.37	2.09	6.64	0	0 to 30.05
	Nutritionist	21	91.3	.045	.158	0	0–.69	0.99	3.47	0	0 to 15.21
	Chiropodist	21	91.3	.002	.006	0	0–.03	0.07	0.25	0	0 to 1.10
	Ambulance	18	78.3	.143	.344	0	0–1.47	14.16	34.05	0	0 to 145.89
	Home enteral nutrition	22	95.7	.045	.216	0	0–1.04	.99	4.76	0	0 to 22.81
Out of home/hospital^a^	GP office visit	18	78.3	.247	.543	0	0–1.75	10.88	23.87	0	0 to 77.00
	Hospital clinic	7	30.4	2.699	4.474	.97	0–20	318.47	527.93	113.93	0 to 2360.00
	Hospice clinic	15	65.2	.536	.809	0	0–2.90	63.27	105.04	0	0 to 341.79
	Clinic (not specified)	20	87.0	.198	.643	0	0–2.95	23.33	75.88	0	0 to 347.79
	Oncologist consultation	18	78.3	.220	.640	0	0–2.95	33.64	97.85	0	0 to 450.95
	Hospice consultant	20	87.0	.205	.606	0	0–2.95	24.15	78.62	0	0 to 347.79
	Hospice nurse	19	82.6	.258	.729	0	0–2.95	3.73	10.57	0	0 to 42.74
	A&E	16	69.6	.273	.643	0	0–2.95	38.40	90.29	0	0 to 414.37
	Hospital nights	14	60.9	1.323	.642	0	0–15.71	490.92	1349.31	0	0 to 5827.41
	Hospice overnights	17	73.9	1.449	3.637	0	0–12.88	537.73	1275.23	0	0 to 4778.48
Medication, supplies (self-paid costs, £)^b^	OTC, food supplement	12	52.2	n/a	n/a	n/a	n/a	3.96	8.36	0	0 to 37.58
	Continence, sheets	16	69.6	n/a	n/a	n/a	n/a	2.41	6.90	0	0 to 32.01
Informal caring (h)	Primary carer	0	0	391.2	228.7	368.7	18.7–672.0	7824	4574	7373	373–13,440
	Secondary carer	14	60.9	122.9	104.5	71.9	0–366	2459	2090	1473	0–6720

^a^Tests: carers reported that seven patients received tests, most frequently blood tests

^b^Medication costs do not include prescription medications as all participants received these free (data on what medications were prescribed were not gathered). No cost was provided for many of the supplies that were reported because they were provided free of charge, e.g. mattresses, hospital bed, speech and language supplies, cushions. One person reported purchasing a disabled car for £4000—including this in the calculation raises the mean (SD) to £86.26 (402.2)

*OTC* over the counter

*GP* general practitioner

Informal caring reported by the primary carer ranged from 1 to 24 h per day and was not significantly correlated with reported burden. Almost 40% of carers reported having a second person to assist with the caring responsibilities. Over a 28-day period, the mean (median) total cost of the primary and supplementary (secondary) informal carer hours, based on replacement cost method using the hourly rate of a clinical support worker, was £10,389 (£10,360).

## Discussion

This pilot study explored potential for using the AHCR in the UK for capturing service use at end-of-life in preparation for a larger observational study to compare models of palliative care. Amongst a variety of different methods used to assess palliative care service use, many of which are specific to individual projects and not comparable or comprehensive, the AHCR has been identified as a validated framework [10]. In assessing the feasibility of using an adapted version of the AHCR as an instrument for collecting formal and informal care costs at the end-of-life, we considered measures of recruitment and retention, acceptability and usability of the data.

*Recruitment* of family carers to this study was problematic and we had a lower enrolment rate (11.9%) than has been reported in previous studies carried out in Canada which used the AHCR (41.8%, 70.4%) [14, 29] and another similar study in the UK (28.5%) [30]. Only one of the six primary care practices that agreed to recruit for the study sent out invitation letters to carers. This low follow-through rate calls into question the suitability of primary care practices as a setting for recruiting participants for palliative care studies. It should be noted that this project took place around the same time as the implementation of centrally imposed changes to primary care (Health and Social Care Act 2012) which may have absorbed primary care practice attention and resources. The one primary care practice that did participate had a research nurse funded by the National Institute of Health Research Clinical Research Network. The hospice had dedicated a staff member to identify patients as part of the parent project which was to evaluate its new telephone navigation service. Such a low recruitment rate raises concerns about research costs and study delays and creates doubts about the representativeness of the samples, suggesting alternative approaches may need to be considered. Lessons may also be drawn from Canadian studies in which palliative care team staff recruited participants direct from their caseload by telephone, rather than by mail as was done here [14, 29]. Recruitment might therefore be more successful with a direct contact from the provider who should have ring-fenced resources to support the recruitment process.

Once recruited, the *retention* rate (70%) was reasonable, given the sensitive nature and timing of data collection, and provides an indirect endorsement of acceptability. Participants who were retained in the study indicated that they were comfortable with the questions asked, indicating good *acceptability* of the adapted AHCR. For some carers, the opportunity to talk about their experiences seemed to be therapeutic, though this required additional time to collect the data and not all commentary related to AHCR questions. Carers frequently contextualised their answers rather than just giving numerical responses. This quite often gave insight into how and why patients and carers used the services they did and also helped to enhance the completeness of the data. Though not the intention of the tool, the two-weekly telephone interview approach produced useful chronological qualitative data on the patient and carer experience of the services used.

The AHCR proved successful for data collection and yielded *complete and usable data* on most items. Missing information was minimal due to the survey interview format and the researcher having a good understanding of how services operate and investigating any discrepancies in carer accounts. As might be expected, informal caring costs, using the replacement cost method, were very high confirming the importance of including this element in evaluative studies and the saving that informal carers afford the health care system. Debate exists, however, about applicability of using replacement methods; if carers gain utility from caring, the costs associated with this should be lower [8, 9].

Many service use items were not widely used, such as counselling and home enteral nutrition, so it might be possible to reduce the burden on carers by only asking them to recall-selected, high cost or commonly used services. Whereas visits inside and outside the home were more easily recalled, phone calls were most difficult for carers to recall and so were not included in analysis due to lack of confidence in the data. We did not ask carers to report the number of prescription medications used as they did not pay out-of-pocket towards them but would choose to do so in the future study for understanding the full cost to the system. Issues with recall may be reduced by distributing an *aide memoire* to carers to remind them of questions of interest and as a place to record service use, particularly as the carers who used a diary seemed to have an easier time reporting their service use at the two-weekly survey interview. Additionally, we would propose changes to the AHCR tool to create a better structure for recording data regarding equipment and supplies on loan as these were recorded as free text but could be structured to make for easier analysis following data collection.

### Strengths and limitations

The strength of the study is in its conception as a pilot study to assess the acceptability of the AHCR method and usability of data generated in a palliative care patient group prior to a larger observational study and recognition of the need for cultural adaptation of the tool before wide use in the UK. In this regard, the study provided useful messages around recruitment and retention, and the costs and benefits of data gathering by telephone survey, to inform the design of future studies in this area. This study achieved a smaller sample size than was planned (23 analysed cases vs 30 planned). However, the study yielded sufficient information to improve the overall process of using the AHCR for the larger, follow-up study. We did not check the accuracy of self-reported service use with records kept by the health system, and for some carers, we perceived that they gave incomplete responses to more complex events, like hospital admission. However, this seemed to be related to the structure of the questions rather than carer recall, as additional probing by an experienced researcher usually provided the required information. We believe that having a qualitatively trained researcher with experience of the palliative care setting administering the questionnaire improved the quality of the data because she could ask appropriate clarifying questions. Alternatively, we could have tested the AHCR against a self-report diary to see which would provide greater recruitment, retention and completeness of data balanced against the research cost of implementing the method.

## Conclusion

The study confirms that the AHCR is a feasible instrument for collecting data on palliative care service use and informal caring costs, though refinement of the recruitment process is needed to make it practical for research purposes. An advantage is that data are gathered regularly thereby reducing the risk of recall errors relative to approaches that contact the carer several months after the patient has died. This improvement in data quality needs to be balanced against the costs of multiple telephone surveys. The focus of the AHCR is on gathering service-use data and understanding the costs of palliative care from a system and societal perspective. Such data have historically been difficult to obtain but are important elements for robust service delivery evaluation.

## Additional files

Additional file 1: UK adapted version of the Ambulatory and Home Care Record (AHCR). (DOCX 48 kb)

Additional file 2: Unit costs used in the costing analysis. (DOCX 14 kb)

## Abbreviations

AHCR: Ambulatory and Home Care Record
GP: General practitioner
