# Supplementary material for: Adaptation of the Ambulatory and Home Care Record for collecting palliative care service utilisation data from family carers in the UK: a pilot study
Source: Pilot Feasibility Stud. 2018 Aug 18;4:141. doi: 10.1186/s40814-018-0332-2 (PMC6098633; doi:10.1186/s40814-018-0332-2)
Supplement: Supplementary file 1 — UK adapted version of the Ambulatory and Home Care Record (AHCR). (DOCX 48 kb) [file 40814_2018_332_MOESM1_ESM.docx]

**Additional file 1**

UK adapted version of the Ambulatory and Home Care Record (AHCR)

Original version: Guerriere DM, Unger WJ, Corey M, et al. Evaluation of the ambulatory and home care record: agreement between self reports and administrative data. *Int J Tech Assess Health Care* 2006; 22(2): 203-210).

**Ambulatory and Home Care Record**

**TELEPHONE SCRIPT & DATA COLLECTION FORM**

**UK version**

Interviewer:_______________________ Patient name: _______________________ ­

Date of Interview:__________________ Carer name: _______________________

**SCRIPT:** Hello this is [interviewer] calling from [organization]. I’ve received your consent form to participate in a telephone interview about *“the patient’s”* experience of health care services. How is [the patient] doing? [*pause*] Are you still able to do the interview at this time?

*If yes, proceed to next section, if no, reschedule interview.*

**SCRIPT:** As you will recall, the purpose of the interview today is to collect information in order to gain a better understanding of the care that patients and their families receive at home and in the hospital or hospice. This interview will take about 15 minutes. Please let me know if you need to end the interview early at any time, or if you find that the questions are becoming too stressful for you.

**INTERVIEW PERIOD**

| **Start date** | **End date (date interview)** |
| --- | --- |
| **Date of death** | **Place of death** |

**CARER AND PATIENT INFORMATION**

*Notes: This section only needs to be completed at time of FIRST interview.*

**SCRIPT:** I’d first like to ask you some background questions about yourself and *“the patient”.*  We’ll start with information about you.

**PRIMARY CARER**

| **Carer ID** | **Male/Female** | **Employment**  *Are you employed? Full time/ part time?* |
| --- | --- | --- |
| **Name** | **Marital status**  *What is your marital status?* | **Age**  *What is your age?* |
| **Relationship to patient**  *What is your relationship to “the patient”?* | **Education**  *What is your highest level of education?*  GCSE or Less  A-Levels  Any Vocation or College  Any Undergraduate  Post Graduate | **Living with patient?**  *Do you live with “the patient”?* |

**SCRIPT:** Now I’d like to ask you some questions about *“the patient”*.

| **Patient name**  *What is his/her name?* | **Age**  *How old is s/he?* | **Male/Female** |
| --- | --- | --- |
| **Diagnosis**  *What is his/her diagnosis?* | **Marital status**  *What is his/her marital status?* | **Living arrangement**  *Does s/he live with anyone?*  lives alone  lives with spouse/partner  lives with children (with or without others)   lives with child  lives with others |
| **Prescriptions**  *Does s/he pay for his/her prescriptions?*  Pay  No pay | **Education**  *What is their highest level of education?*  GCSE or Less  A-Levels  Any Vocation or College  Any Undergraduate  Post Graduate | **Attendance allowance**  *Does s/he receive attendance allowance?*  *Does s/he receive a direct payment or personal budget from social services?* |

**Health and social care visits/appointments inside the home**

**SCRIPT:** I would now like to ask you about events during the last two weeks from [start date] until [today]. If you keep track of the appointments it would be helpful if you could get your diary.

**Notes**

**SCRIPT**: In the [past two weeks], has “*the patient”* had any visits or appointments **inside the home**?

Interviewer notes:

- **Prompt:** Healthcare visits include: district or community nurse, respiratory/cardiac nurse, GP, massage therapist, physiotherapist, hospice community nurse, hospice doctor, Marie Curie nurse, hospice at home team, nurse plus.
- **Prompt:** Social care visits include: carers who help with daily tasks, such as bathing, dressing, food preparation.
- When asking about private funding: Did you have to pay anything out of pocket towards this visit?

If **YES**, ask carer details about each visit – **HOME VISITS**:

**Visit 1**- Provider type: ________________ # of visits:____ Pay? 🞎 No 🞎 Yes £: _____________

**Visit 2**- Provider type: ________________ # of visits:____ Pay? 🞎 No 🞎 Yes £: _____________

**Healthcare visits/appointments outside the home**

**SCRIPT**: In the [past two weeks], has *“the patient”* had any healthcare appointments **outside the home**?

Interviewer notes:

- Includes appointments for tests.
- When asking about private funding: Did you have to pay anything out of pocket towards this visit?

**Visit 1**- Provider type: ______________ First visit: 🞎 Yes 🞎 No # of visits:____ Pay? 🞎 No 🞎 Yes £: _______

Did *“the patient”* also have to have tests done? If yes, type: _____________________________________________

Mode: car/bus ________ Time travelling to appointment: __________ Costs (parking, transportation): _________

**Visit 2**- Provider type: ______________ First visit: 🞎 Yes 🞎 No # of visits:____ Pay? 🞎 No 🞎 Yes £: _______

Did *“the patient”* also have to have tests done? If yes, type: _____________________________________________

Mode: car/bus ________ Time travelling to appointment: __________ Costs (parking, transportation): _________

**A&E, HOSPITAL, HOSPICE**

**SCRIPT:** In the [past two weeks] has *“the patient”* been admitted to the hospital or to A&E?

If **YES**

- How many times did *"the patient"* visit A&E? ___________
- How many days/nights did *"the patient"* spent in hospital? _____________
- How many times did you go to visit them? _____________

**SCRIPT:** In the [past two weeks] has *“the patient”* been admitted to a hospice as an inpatient?

- How many days/nights did *"the patient"* spend in hospice? _____________
- How many times did you go to visit them? _____________

| **# of days in hospital:**  **# of nights in hospital:** | **# of visits to A&E:** | **# of days in hospice:**  **# of nights in hospice:** |
| --- | --- | --- |
| ***1^st^ Stay, inquire about travel***  Number of times visited _____________  Round trip (miles) __________________  Round trip (min) ___________________  Mode transportation _______________  £ Parking _________________________  £ Public transit/taxi | ***1^st^ Visit, inquire about travel***  Round trip (miles) _________________  Round trip (min) ___________________  Mode transportation _______________  £ Parking _________________________  £ Public transit/taxi | ***1^st^ Stay, inquire about travel***  Number of times visited _____________  Round trip (miles) __________________  Round trip (min) ___________________  Mode transportation ________________  £ Parking _________________________  £ Public transit/taxi |
| ***2nd Stay, inquire about travel***  Number of times visited _____________  Round trip (miles) __________________  Round trip (min) ___________________  Mode transportation _______________  £ Parking _________________________  £ Public transit/taxi | ***2^nd^ Visit, inquire about travel***  Round trip (miles) _________________  Round trip (min) ___________________  Mode transportation _______________  £ Parking _________________________  £ Public transit/taxi | ***2^nd^ Stay, inquire about travel***  Number of times visited _____________  Round trip (miles) __________________  Round trip (min) ___________________  Mode transportation ________________  £ Parking _________________________  £ Public transit/taxi |

**TELEPHONE CALLS**

**SCRIPT**: During the past 2 weeks, have you or any other carers spoken with any health or social care professionals on the phone about “*the patient’s*” care?

| **Person spoken to (i.e. type of provider)** | **Average Call Duration** | **# calls during interview period** |
| --- | --- | --- |
|  |  |  |
|  |  |  |
|  |  |  |

**NOTES**

**MEDICATIONS**

If patient pays for prescriptions **SCRIPT:** Did you or a member of your family or friends pick up any prescription medication in the last two weeks which was for *“the patient”?*

**SCRIPT:** Did you or a member of your family or friends purchase any over the counter medicine in the last two weeks which was for *“the patient”?*

**SCRIPT:** Did you or a member of your family or friends purchase any food supplements or alternative medicine for *“the patient”* in the past 2 weeks?

Interview notes:

- This can include new medication or existing drugs that were refilled in past 2 weeks
- Include all items purchased in relation to *“the patients”* health

| **Medication type** | **How much did you pay?** | **Quantity** | *Total cost* | **Comments** |
| --- | --- | --- | --- | --- |
| Prescription  OTC  Food supplement  Other |  |  |  |  |
| Prescription  OTC  Food supplement  Other |  |  |  |  |

**Supplies and Equipment**

**SCRIPT:** Do you know of any medical supplies purchased or received in the last two weeks for *“the patient”*?

Interview notes:

- **Prompt:** Incontinence products, bathroom railings, bandages, thermometers, wheelchair/walkers, catheters, IV bags. Amount paid.

Who purchased it and how much did they pay

| **Supply** | **Quantity** | **Purchased by** |  |
| --- | --- | --- | --- |
|  |  | - Paid by ____________________________ - Free - Borrowed | **Out of pocket cost:** |
|  |  |  |  |
|  |  |  | **Borrowed from:** |
|  |  | - Paid by ____________________________ - Free - Borrowed | **Out of pocket cost:** |
|  |  |  |  |
|  |  |  | **Borrowed from:** |

**Notes:**

|  |
| --- |

**CARE GIVING EPISODES**

**SCRIPT:** Can you tell me how much time you have spent looking after *“the patient”* in the last two weeks?

Interviewer notes:

- Time off work/sick/holiday/unpaid
- Collect demographic information before moving on to other carers

**PRIMARY CARER**

**TIME SPENT CAREGIVING**

| **Carer ID** |  |  |
| --- | --- | --- |
| **# Hours: on average per day in last 2 weeks** | **24 hour care provided: Y / N**  *Do not ask this question, indicate yes only if the respondent volunteers they provided care 24/7.* | **# holiday hours** |
| **On leave from work for caring?** | **Notes** | |

**SCRIPT:** Can you tell me how much time friends and family have spent looking after “*the patient”* in the last two weeks (who were not paid to do so)?

Interviewer notes:

- Amount of hours
- Age & Sex of carer; Working Pattern (i.e. did they take time off work /sick/holiday/unpaid)

**OTHER CARERS (complete for all unpaid carers)**

| **Carer name** | **Male/Female** | **Age** | **Relationship to patient** |
| --- | --- | --- | --- |
| **Carer ID** | **24 hour care provided: Y / N**  *Do not ask this question, indicate yes only if the respondent volunteers they provided care 24/7.* | | **# holiday hours** |
| **# Hours:** | **On leave from work for caring?** | | **Notes** |

| **Carer name** | **Male/Female** | **Age** | **Relationship to patient** |
| --- | --- | --- | --- |
| **Carer ID** | **24 hour care provided: Y / N**  *Do not ask this question, indicate yes only if the respondent volunteers they provided care 24/7.* | | **# holiday hours** |
| **# Hours:** | **On leave from work for caring?** | | **Notes** |

**The following sections were added to the end of the data collection tool, but are not a part of the AHCR:**

**Patient symptom and functional assessment using the Eastern Cooperative Oncology Group scale** (Oken MM, Creech RH, Tormey DC, et al. Toxicity and response criteria of the Eastern Cooperative Oncology Group. *Am J Clin Oncol* 1982; 5:649-655.

**Caregiver’s burden scale in end of life** (Dumont S, Fillion L, Gagnon P, et al. A new tool to assess family caregivers’ burden during end-of-life care. *J Palliat Care* 2008; 24(3):151-161.

**Satisfaction with services:** Please could you tell me if the services received: exceeded your expectations / just met your expectations/ or fell short of your expectations?
