# Supplementary material for: Adaptation of the Ambulatory and Home Care Record for collecting palliative care service utilisation data from family carers in the UK: a pilot study
Source: Pilot Feasibility Stud. 2018 Aug 18;4:141. doi: 10.1186/s40814-018-0332-2 (PMC6098633; doi:10.1186/s40814-018-0332-2)
Supplement: Supplementary file 2 — Unit costs used in the costing analysis. (DOCX 14 kb) [file 40814_2018_332_MOESM2_ESM.docx]

**Additional file 2**

Unit costs used in the costing analysis

***Sources:*** *PSSRU Unit Costs of Health and Social Care 2015 and NHS National Schedule of Reference Costs 2014-2015*

| **Service** | | **Unit Cost** | **Source** |
| --- | --- | --- | --- |
| In home | General practitioner visit | £70.75/visit | PSSRU sections 10.8a and 10.8b General practitioner – cost elements and unit costs resp. - £225 per hour of patient contact for 11.4 minutes, length of a home visit consultation, plus £140 per hour of GMS activity for 12 minutes, average travel time per home visit. |
|  | District nurse visit  Hospice Nurse visit  Hospice outreach | £27.30/visit | PSSRU section 10.1 Community nurse - £67 per hour of patient-related work lasting 15.5 minutes, assumed consultation length, plus £50 per hour lasting 12 minutes, assumed travel time. |
|  | Home care, arranged by social services | £12/visit | PSSRU section 11.6 Home care worker - £24 per hour weekday of face-to-face contact, assuming 30 minutes duration per visit |
|  | Counsellor visit | £42/use | PSSRU section 12.1 NHS community mental health team (CMHT) for older people with mental health problems - £42 per hour per team member where duration of a visit is 60 minutes. |
|  | Occupational therapist visit  Physiotherapist visit  Nutritionist visit  Home Enteral Nutrition (N+HEN) | £22/visit | PSSRU section 11.5 Community occupational therapist (local authority) - £44 per hour lasting an assumed consultation length of 30 minutes. |
|  | Chiropodist visit | £40/visit | NHS Reference costs – Service code AHP, Currency code A09A (Podiatrist, Tier 1, General Podiatry). Use national average unit cost of £40 |
|  | Ambulance | £99/use | PSSRU section 7.1 NHS reference costs for hospital services – National average of all ambulance services (£99) |
| Out of home/ hospital/ hospice | GP office visit | £44/visit | PSSRU section 10.8b General practitioner – unit costs - £44 per patient contact lasting 11.7 minutes, length of a consultation at surgery. |
|  | Hospital clinic visit  Hospice clinic visit  Clinic visit not specified | £118/visit | PSSRU section 7.1 NHS reference costs for hospital services – National average of consultant-led outpatient attendances (£118) |
|  | Oncologist consultation  Hospice consultant | £153 / consultation | PSSRU section 7.1 NHS reference costs for hospital services – National average of specialist palliative care consultant-led outpatient attendances |
|  | Hospice nurse | £14.50/visit | PSSRU section 10.6 Nurse (GP practice) - £56 per hour of face-to-face contact lasting 15.5 minutes, duration of surgery consultation. |
|  | Visit A&E but not admitted | £140.59/visit | NHS Reference Costs – Total Outpatient Attendances, Service code 180 (Accident & Emergency). Use ‘total’ unit cost of £140.69. |
|  | In-hospital stay overnight  Hospice stay overnight | £371 / night | PSSRU section 7.1 NHS reference costs for hospital services – Inpatient – specialist palliative care (adults only), average cost per bed day |
| Informal caring | Primary or secondary informal carer, hours per day | £20 / hour | PSSRU section 10.5, Clinical support worker |

Curtis L and Burns A. Unit costs of health and social care 2015. Report. Canterbury: Personal and Social Services Research Unit
